# Pesticide detection in postmortem serum, hair, and cerebrospinal fluid from cases of sudden unexpected death in infancy: a registry-based study

**DOI:** 10.1007/s00431-026-07546-6

**Published:** 2026-10-24

**Authors:** Emmanuel Bourdet, Pascal Caillet, Fabien Mercier, Jean-Philippe Antignac, Aurélien Lecorgne, Ronan Pommereuil, David Boels, Rémi Béranger

**Affiliations:** 1 Univ Rennes, Inserm, Irset (Institut de Recherche en santé, Environnement et Travail) UMR 1085, Rennes, France; 2 Nantes Université, CHU Nantes, Service de Santé Publique et Environnementale, 44000 Nantes, France; 3 Optimisation Thérapeutique en Neuropharmacologie OTEN U1144, Université Paris-Cité, INSERM, 75006 Paris, France; 4 Univ Rennes, Inserm, EHESP, Irset (Institut de recherche en santé, environnement et travail) - UMR_S 1085, 35000 Rennes, France; 5 France Exposome - Breizh Exposome LERES, French National Infrastructure in Exposomics, Rennes, France; 6 ONIRIS, INRAE, Laberca UMR, Nantes, 1329 France; 7 Univ Rennes, CHU Rennes, Inserm, Irset (Institut de Recherche en santé, Environnement et Travail) UMR 1085, Rennes, France

**Keywords:** Sudden Unexpected Death in Infancy, Pesticide, Environmental exposure, Infant mortality, Biomonitoring

## Abstract

Sudden unexpected death in infancy (SUDI) is the leading cause of death between 1 month and 1 year in France. Its underlying mechanisms and the role of environmental exposures remain poorly understood. Pesticides may theoretically contribute to SUDI through neurotoxic pathways, but biomonitoring studies of SUDI cases are scarce, limiting etiological research. We aimed to assess pesticide exposure in SUDI cases and to explore potential factors associated with exposure across three biological matrices: hair, serum and cerebrospinal fluid (CSF). We analyzed pesticide exposure markers in post-mortem serum samples (*n* = 98), hair (*n* = 57), and CSF (*n* = 97) from SUDI cases in France. Twenty-seven organochlorine pesticides were measured in serum and CSF, and 27 pesticides of various families in hair. We performed clustering based on exposure detection within each matrix and used logistic regression to assess associations between exposure clusters and several factors. Banned but persistent organochlorines, including p,p′-DDE, HCB, and β-HCH, were detected in serum. β-HCH and p,p′-DDE were also present in CSF. Hair samples revealed biomarkers of exposure to chlorpyrifos, fipronil, and cypermethrin/permethrin. Serum clusters with the highest detection were associated with higher birthweight and breastfeeding. CSF clusters with the highest detection were associated with breastfeeding and younger age at death.

*Conclusion*: Although these findings do not yet clarify SUDI risk factors and mechanisms, they support the analytical feasibility of using post-mortem multi-matrix samples for pesticide exposure investigations. The detection of pesticides in the CSF of very young infants warrants further investigation of potential neurotoxicity.

**Table:** 

**What is Known:** • *SUDI remains a leading cause of infant mortality, with largely unknown etiology.* • *Pesticide neurotoxicity is a plausible contributor, but data on pesticide exposure in SUDI cases are scarce.*	
**What is New:** • *Diverse pesticides were detected in the serum, CSF, and hair of SUDI cases.* • *These findings call for etiological studies on SUDI and pesticide exposure.*

**Supplementary information:**

The online version contains supplementary material available at https://doi.org/10.1007/s00431-026-07546-6.

## Introduction

Pesticides are chemicals used to control pests and diseases [1]. Widely used in agriculture, households, and medicine, they represent a significant burden on environmental health [2]. Consequently, exposure sources are diverse, ranging from food and water to proximity to treated crops or occupational and domestic use [3]. Exposure is ubiquitous, involving even persistent organic pollutants banned under the Stockholm Convention. Numerous studies have linked pesticide exposure to adverse child and fetal health outcomes, including cancer, neurodevelopmental disorders, endocrine disruption, spontaneous abortion, congenital malformations, low birthweight, stillbirth, and fetal death [4–6].

Sudden unexpected death in infancy (SUDI) is defined as the sudden, unexpected death of an infant with no obvious cause prior to investigation [7]. SUDI remains a major cause of infant mortality in many countries, and the cause often remains unknown despite postmortem investigation. While known risk factors include prone sleeping position, preterm birth, and tobacco exposure, the specific etiology remains unexplained in most cases [8]. Given the potential neurotoxic mechanisms implicated in SUDI [9], pesticides—known for their neurotoxicity, including alteration of autonomic nervous system (ANS) neurotransmitters [10, 11]—could contribute to these deaths. In 2025, we conducted a systematic literature review on SUDI and pesticide exposure [12]. Although we found some evidence of an association, inconsistent exposure assessment, unclear health categorization, low statistical power, and limited biomarkers prevented firm conclusions.

Assessing the role of pesticides in SUDI presents significant methodological challenges. While postmortem samples from SUDI cases may be available, obtaining comparable samples from infant controls is difficult. Studies on pesticide exposure in infant populations are scarce, precluding strong hypotheses about likely detection levels. The presence of pesticides in the nervous system of infants remains poorly studied due to the invasiveness of the sample collection, with the exception of few studies detecting organochlorines and organophosphates in postmortem brain samples [13, 14]. Assessing pesticide detections in SUDI biological samples is therefore essential before a large etiological study. Matrices of interest include serum (the standard for persistent substances), hair (non-invasive and cumulative), and cerebrospinal fluid (CSF), which reflects direct exposure of the nervous system. A descriptive exposure study may also help to identify potential factors associated with exposure.

The aim of this exploratory study was to assess pesticide exposure in SUDI cases using serum, CSF and hair samples, and to investigate associations between exposure clusters and several cofactors.

## Materials and methods

### Study design and population

**Fig. 1:**
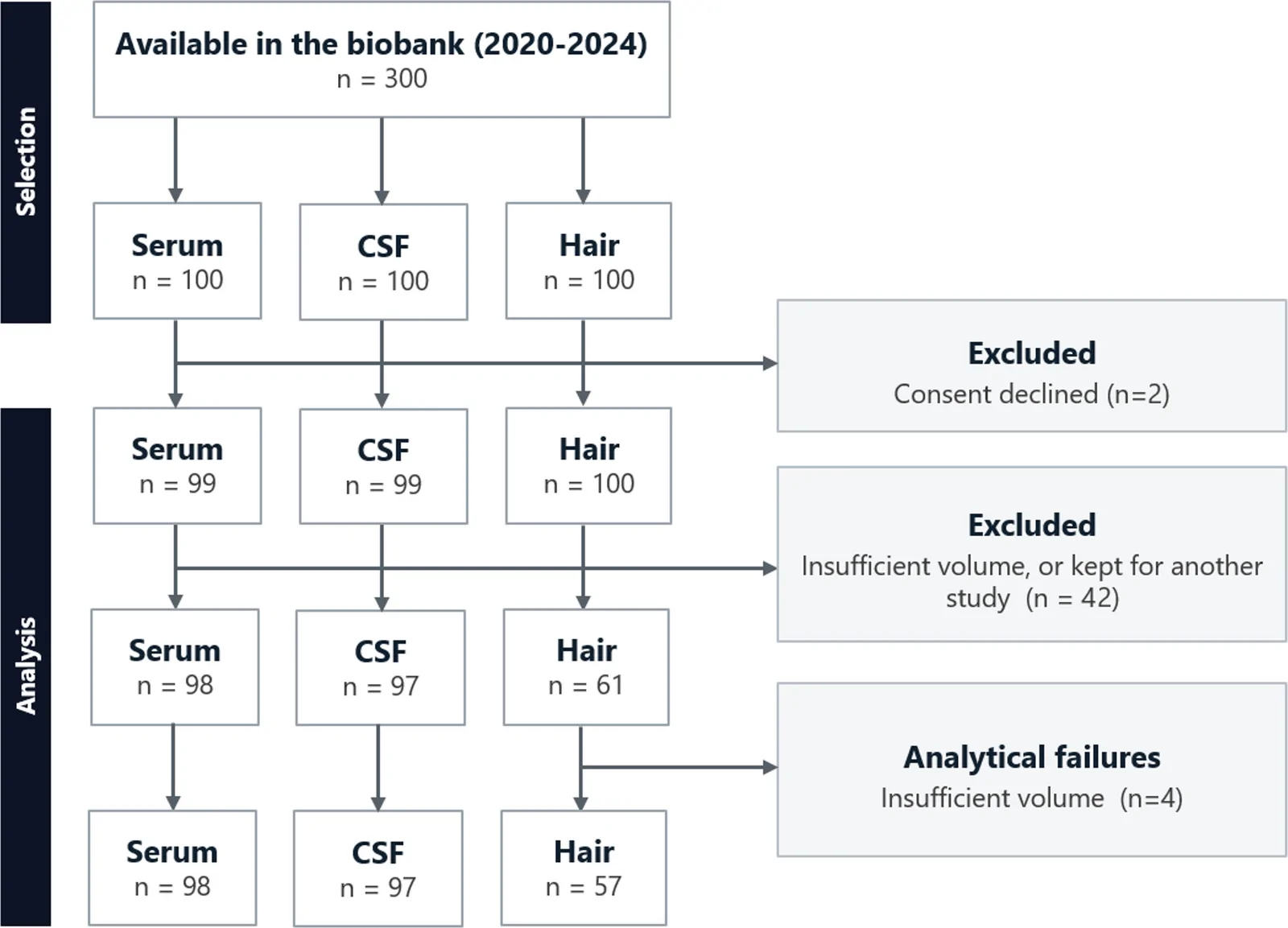
Flowchart for sample selection

The French SUDI registry (*Observatoire de la Mort Inattendue du Nourrisson, OMIN*) has collected data since 2016 and biological postmortem samples since 2020 from SUDI cases (up to 24 months of age) in France [15, 16]. Serum, CSF and hair samples included in our study met the following criteria: inclusion in the registry (i.e., meeting the French definition of SUDI up to 24 months of age and residence in continental or overseas French territories) and sample availability. Two exclusion criteria were applied: insufficient sample volume (less than 20 mg for hair, 500 µl for serum, and 200 µl for CSF) and refusal to give parental consent (Fig. 1).

We analyzed 98 serum, 97 CSF, and 57 hair samples from 168 infants who died between 2020 and 2024. All three matrices were available for 14 infants. The remaining cases had one or two matrices depending on sample availability and volume (Table 1).

**Table 1:** Number of infants for whom data for one, two or three biological matrices were available

Serum	Hair	CSF	Number of infants (*n*)
Available	Available	Available	14
	Available	Available	12
Available	Available		13
Available		Available	31
		Available	40
	Available		18
Available			40

Analyses were conducted with parental consent and approval from the registry’s scientific council, the national ethics committee (CESREES), and the French data protection authority (CNIL) (Approval # 924,124).

Details on sampling methods are provided in the Supplementary Material.

### Selection of the target pesticides

Given the exploratory nature of this study, we performed targeted analyses and we prioritized pesticide families based on their detection rates in a previous biomonitoring study in pregnant women in France [17], probable toxicity [4], and data from the Comparative Toxicogenomics Database (https://ctdbase.org/). For serum and CSF, we analyzed 27 organochlorine compounds (OCs); for hair, we analyzed four OCs, seven organophosphates (OPs), 11 pyrethroids (PYRs), two phenylpyrazoles (PAs), one carbamate (CA), one neonicotinoid (NC), and one pyrethroid adjuvant (A-PYR) (Table 3). We focused on OCs in serum and CSF given their persistence and established role as markers of long-term exposure.

### Chemical analyses

Hair samples were analyzed by LERES (Rennes, France) and serum/CSF by LABERCA (Nantes, France). For hair, the entire sample was analyzed due to limited mass and difficulty identifying the root. The samples were not washed to avoid uncontrolled variability [18]. Hair samples were pulverized prior to extraction using acetonitrile. Hair, serum and CSF samples were all analyzed by gas chromatography and tandem mass spectrometry (GC/MS/MS). A detailed description of the analytical methods is provided in the Supplementary Material.

### Statistical analysis

We described demographic and clinical characteristics of the study population and summarized pesticide exposure by detection frequency above the limits of detection (LOD). Because our approach is exploratory, we did not use limits of quantification (LOQ) to avoid masking certain detections, and because LOD is better suited to categorical analyses.

For substances with high detection rates, distributions (25th, 50th, and 75th percentiles) were also calculated. Pesticides with detection rates < 10% were excluded. The remaining pesticides were classified based on detection frequency, following precedent studies [17, 19]:

- Detection < 50%: two classes (< LOD; > LOD)
- Detection 50–69%: three classes (< LOD; between the LOD and median; > median)
- Detection 70–89%: four classes (< LOD; 1st tertile; 2nd tertile; 3rd tertile)
- Detection ≥ 90%: four quartiles (with the 1st quartile including data < LOD).

Polychoric correlation analyses were performed on these ordered classes to visualize associations. As not all SUDI cases had all three matrices, classifications were performed separately for serum, CSF, and hair. Inter-matrix relationships were explored using overlapping datasets (pairwise and all three combined).

Factorial analysis (Multiple Correspondence Analysis, MCA) and hierarchical clustering (HCPC) were used to identify exposure profiles [20, 21]. Clusters were defined solely by exposure data. The number of clusters was determined by their position on the factorial map and the inertia gain graph. We then examined the association between categorical cofactors and exposure clusters using logistic regression. Cofactors included binary data: age at death (0–2 months; ≥ 3 months), birthweight (< 2500 g; ≥ 2500 g), position at discovery (prone; other), biological sex (male; female), breastfeeding (yes; no), preterm birth (yes; no), and the French deprivation index (Fdep) in quintiles (1st quintile = wealthiest areas). All data, except pesticide exposure, were provided by the French SUDI registry. More details on cofactors and regression methods are provided in the Supplementary Material.

All analyses were performed with RStudio software (version 4.1). A short example of R code is provided in the Supplementary Material.

### Sensitivity analyses

In MCA and HCPC analyses, the number of categories per variable can influence results: variables with two categories (detection < 50%) and those with four categories (detection > 70%) contribute differently, potentially biasing results. We therefore performed a supplementary analysis within each classification profile (i.e., including only variables with two categories) to assess pattern stability and identify potentially spurious associations. For both age and birthweight, we used continuous data to perform linear regression and compare results with the main analysis.

### Missing data

Data were missing for several variables. Although we had planned to analyze 100 samples per matrix, we had access to only a limited number of eligible samples, owing to the recent establishment of the registry biobank (since 2020), sample use in other studies, limited volume, and lack of consent. Hair samples were particularly difficult to collect, given limited quantity in young children and the need to preserve the infant’s appearance during the postmortem process. Data were missing for several factors, particularly hair clusters (Table 2), and were presented as unknown in the descriptive analysis. In the cluster-based regression, we did not perform imputation, as our approach remained essentially descriptive. Unknown data were excluded.

**Table 2:** Characteristics of the infants (*n* = 168)

		*n*	%	Mean (SD)	min:max	p25	Median	p75
Age at death (months)				5.7 (6.1)	0:23	1	4	8
	0–2 months	67	39.9					
	≥ 3 months	101	60.1					
Birthweight (g)				2882.8 (728.9)	924:4470	2474	3002	3406
	< 2,500 g	36	21.4					
	≥ 2,500 g	104	61.9					
	Unknown	28	16.7					
Sex								
	Female	78	46.4					
	Male	90	53.6					
Position								
	Prone	22	13.1					
	Other	128	76.2					
	Unknown	18	10.7					
Year of death								
	2020	10	6.0					
	2021	20	11.9					
	2022	56	33.3					
	2023	65	38.7					
	2024	17	10.1					
Breastfeeding								
	No	79	47.0					
	Yes	58	34.5					
	Unknown	31	18.5					
Fdep								
	1 st quintile	24	14.3					
	2nd quintile	27	16.1					
	3rd quintile	29	17.2					
	4th quintile	31	18.5					
	5th quintile	34	20.2					
	Unknown	23	13.7					
Preterm birth								
	No	107	63.7					
	Yes	34	20.2					
	Unknown	27	16.1					

*Fdep*, French deprivation index

## Results

### Characteristics of the infants included

Among the 168 infants included, the median age at death was 4 months; 39.9% died between 0 and 2 months. Most were male (53.6%) and found in a position other than prone (76.2%). 21.4% of infants had a low birthweight (< 2500 g), 34.5% were breastfed, and 20.2% were born prematurely. Deaths occurred primarily in 2023 (38.7%) and 2022 (33.3%). Regarding socioeconomic status, 20.2% of infants lived in poorer areas (5th quintile) versus 14.3% in the wealthiest areas (1st quintile) (Table 2).

### Exposure assessment

Tables 3 and 4 summarize pesticide detection frequencies. In serum, the most frequent detections were p,p′-DDE (100%), HCB (71.4%), β-HCH (70.4%), *cis*-heptachlor epoxide (68.4%), and p,p′-DDT (51%). CSF analysis detected particularly p,p′-DDE (15.5%) and β-HCH (12.4%). In hair, three organochlorines were detected: γ-HCH (lindane, 91.2%), p,p′-DDE (42.1%), and HCB (7.0%). Ten additional compounds exceeded 10% detection frequency in hair, notably fipronil sulfone (57.9%), TCPy (56.1%), and cypermethrin (56.1%).

**Table 3:** Detection of organochlorine pesticides in serum (*n* = 98) and CSF (*n* = 97) samples from the French SUDI registry

Substance	Serum results (pg/g) *n*= 98					CSF results (pg/g) *n*= 97				
	LOD (min–max)	Detection (%)	p25	Median	p75	LOD (min–max)	Detection (%)	p25	Median	p75
p,p′-DDE	< 1.92	100.0	15.81	32.06	88.29	1.31–10.51	15.5	< LOD	< LOD	< LOD
HCB	4.3–11.33	71.4	< LOD	9.64	15.25	4.06–29.78	3.1	< LOD	< LOD	< LOD
β-HCH	0.89–3.43	70.4	< LOD	3.8	8.68	0.67–7.66	12.4	< LOD	< LOD	< LOD
*Cis*-heptachlor epoxide	2.78–5.12	68.4	< LOD	5.14	12.0	2.82–22.09	1.0	< LOD	< LOD	< LOD
p,p′-DDT	2.06–4.94	51.0	< LOD	2.88	6.87	2.14–15.73	1.0	< LOD	< LOD	< LOD
Dieldrin	6.83–22.03	33.7	< LOD	< LOD	13.92	5.73–86.11	0			
*Trans*-nonachlor	3.93–8.77	24.5	< LOD	< LOD	< LOD	4.69–37.94	0			
p,p′-DDD	1.17–3.31	24.5	< LOD	< LOD	< LOD	1.89–14.49	0			
*Cis*-nonachlor	1.47–3.2	9.2	< LOD	< LOD	< LOD	1.77–14.51	0			
o,p′-DDD	0.53–1.31	6.1	< LOD	< LOD	< LOD	0.93–7.37	0			
o,p′-DDT	2.33–5.31	5.1	< LOD	< LOD	< LOD	2.85–21.58	0			
Oxychlordane	7.96–17.21	4.1	< LOD	< LOD	< LOD	9.68–71.53	0			
γ-HCH (lindane)	13.97–38.7	3.1	< LOD	< LOD	< LOD	15.65–122.4	0			
α-HCH	7.96–16.15	1.0	< LOD	< LOD	< LOD	8.9–66.74	0			
δ-HCH	3.45–10.7	1.0	< LOD	< LOD	< LOD	4.12–30.25	0			
PeCBz	3.26–9.36	0				3.48–25.57	0			
Heptachlor	1.16–2.93	0				1.09–9.93	0			
Aldrin	2.16–5.42	0				1.96–30.94	0			
*Trans*-heptachlor epoxide	6.65–13.9	0				7.52–55.2	0			
o,p′-DDE	1.18–2.8	0				1.89–14.89	0			
γ -Chlordane	1.44–8.29	0				1.84–14.25	0			
α-Endosulfan	37.51–116.6	0				43.32–331.2	0			
α-Chlordane	3.97–8.26	0				4.79–37.48	0			
Endrin	6.79–22.48	0				8.51–91.41	0			
β-Endosulfan	42.12–129.8	0				46.65–417.9	0			
Endosulfan sulfate	19.16–48.2	0				22.13–181.4	0			
Mirex	1.72–8.55	0				4.43–34.79	0			

*DDD* dichlorodiphenyldichloroethane, *DDE* dichlorodiphenyldichloroethylene, *DDT* dichlorodiphenyltrichloroethane, *HCH*, hexachlorocyclohexane, *HCB* hexachlorobenzene, *PeCBz*, pentachlorobenzene. As the LOD varied between samples, “LOD (min-max)” represents the range of LOD values obtained in our analyses

**Table 4:** Detection of pesticides in hair (ng/g, *n* = 57) samples from the French SUDI registry

Family	Substance	LOD (min–max)	Detection (%)	p25	Median	p75
OC	γ-HCH (lindane)	0.2–0.42	91.2	0.53	0.88	1.21
PA	Fipronil sulfone	0.2–0.42	57.9	< LOD	0.37	0.71
OP	TCPy	0.98–2.08	56.1	< LOD	1.12	1.77
PYR	Cypermethrin	0.98–2.08	56.1	< LOD	2.47	10.05
OC	p,p′-DDE	0.2–0.42	42.1	< LOD	< LOD	0.34
PYR	Permethrin	49–104.06	36.8	< LOD	< LOD	103
PYR	*Trans*-Cl2CA	0.98–2.08	35.1	< LOD	< LOD	2.29
PYR	Transfluthrin	0.98–2.08	31.6	< LOD	< LOD	1.06
A-PYR	BPO	9.8–20.81	28.1	< LOD	< LOD	10.4
PYR	*Cis*-Cl2CA	0.98–2.08	24.6	< LOD	< LOD	< LOD
PA	Fipronil desulfinyl	0.2–0.42	15.8	< LOD	< LOD	< LOD
CA	Propoxur	0.98–2.08	12.3	< LOD	< LOD	< LOD
PYR	Deltamethrin	0.98–2.08	8.8	< LOD	< LOD	< LOD
PYR	Tetramethrin	9.8–20.81	8.8	< LOD	< LOD	< LOD
OC	HCB	0.2–0.42	7.0	< LOD	< LOD	< LOD
NN	Acetamiprid	4.9–10.41	5.3	< LOD	< LOD	< LOD
PYR	*Cis*-ClCF3CA	4.9–10.41	5.3	< LOD	< LOD	< LOD
PYR	*Cis*-Br2CA	0.98–2.08	3.5	< LOD	< LOD	< LOD
PYR	D-Phenothrin	49–104.06	3.5	< LOD	< LOD	< LOD
OP	IMHP	4.9–10.41	1.8	< LOD	< LOD	< LOD
OP	Diazinon	4.9–10.41	0			
OP	Malathion	0.98–2.08	0			
OP	Fosthiazate	4.9–10.41	0			
OP	Phorate	4.9–10.41	0			
OP	Dimethoate	4.9–10.41	0			
OC	β-HCH	0.98–2.08	0			
PYR	Cyfluthrin	0.98–2.08	0			

As the LOD varied between samples, “LOD (min–max)” represents the range of LOD values obtained in our analyses

*OC *organochlorine, *OP *organophosphate, *PYR *pyrethroid, *PA* phenylpyrazole, *CA *carbamate, *A-PYR* pyrethroid adjuvants, *NC *neonicotinoid, *DDD *dichlorodiphenyldichloroethane, *DDE* dichlorodiphenyldichloroethylene, *DDT *dichlorodiphenyltrichloroethane, *HCH *hexachlorocyclohexane, *HCB *hexachlorobenzene, *PeCBz* pentachlorobenzene, *cis-Br2CA *cis-3-(2,2dibromovinyl)−2,2-dimethylcyclopropane-carboxylic acid, *cis-ClCF3CA *3-(2-chloro-3,3,3-trifluoro-1-propenyl)−2,2-dimethylcyclopropanecarboxylic acid, *IMPH* 2‑isopropyl‑4‑methyl‑6‑hydroxypyrimidine, *Cl2CA *cis-3-(2,2dichlorovinyl)−2,2-dimethylcyclopropane-carboxylic acid, *BPO* benzoyl peroxide, *TCPy* 3,5,6-trichloro-2-pyridinol

### Correlation analyses

**Fig. 2:**
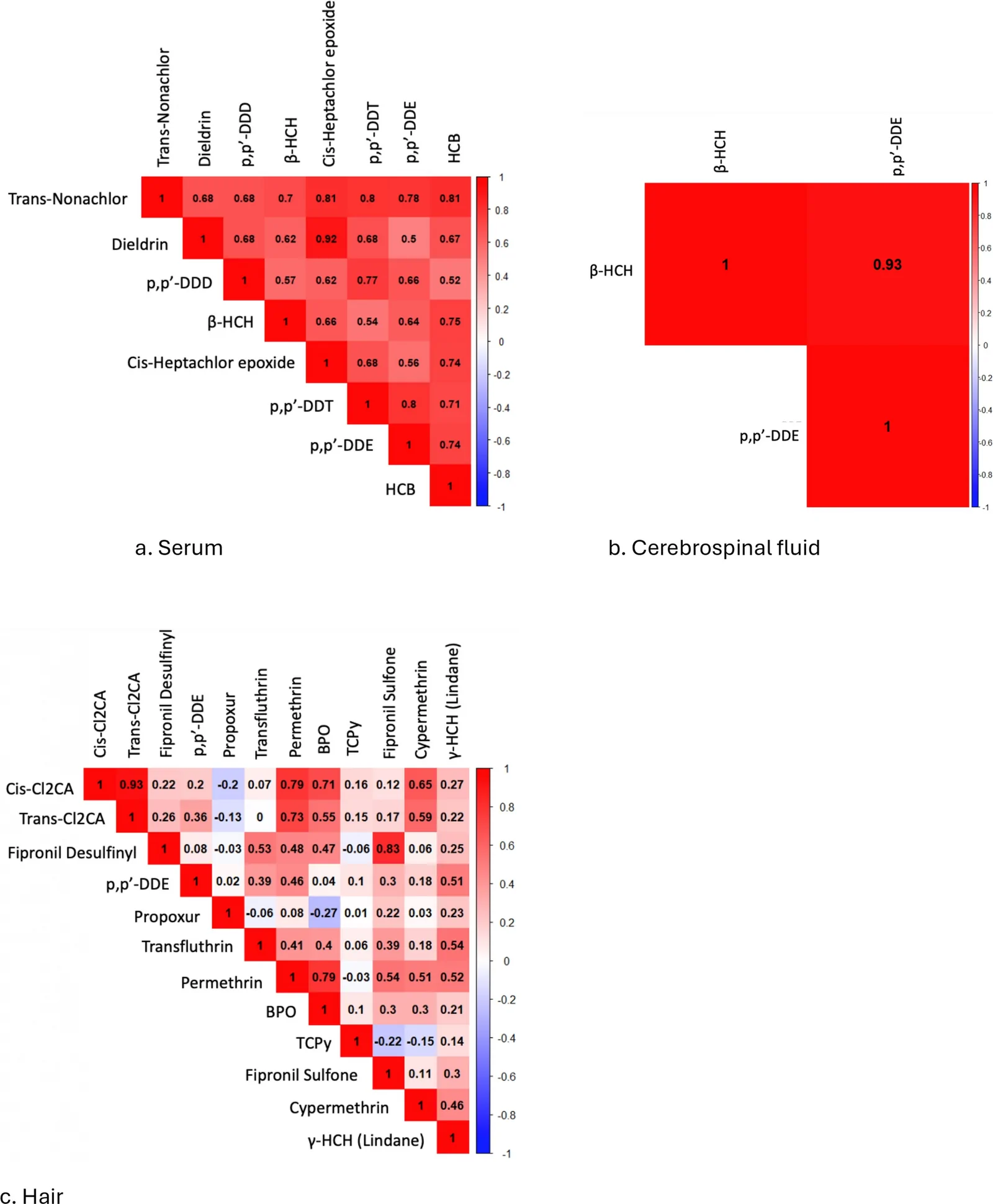
Polychoric correlation results between pesticides detected in more than 10% of the samples for each matrix (**a** serum, **b** cerebrospinal fluid, **c** hair). Abbreviations: CSF, cerebrospinal fluid; DDD, dichlorodiphenyldichloroethane; DDE, dichlorodiphenyldichloroethylene; DDT, dichlorodiphenyltrichloroethane; HCH, hexachlorocyclohexane; HCB, hexachlorobenzene; Cl2CA, cis-3-(2,2dichlorovinyl)-2,2- dimethylcyclopropane-carboxylic acid; BPO, benzoyl peroxide; TCPy, 3,5,6-trichloro-2-pyridinol. The color key ranges from red for very strong positive correlations to blue for strong negative correlations. Pink and pale blue indicate moderate positive and negative correlations, respectively. Blank indicates no correlation

Within-matrix correlations between pesticides were strongest in serum (coefficients > 0.5), particularly between p,p′-DDE and p,p′-DDT (0.80), and between dieldrin and *cis*-heptachlor epoxide (0.92). In CSF, only p,p′-DDE and β-HCH were detectable, and highly correlated (0.93). Hair correlations were variable. Metabolites of the same parent compound (e.g., *cis*-Cl2CA/*trans*-Cl2CA; fipronil desulfinyl/fipronil sulfone) were highly correlated (0.93 and 0.83, respectively). Pyrethroid metabolites (*cis*-Cl2CA, *trans*-Cl2CA) and the synergist BPO were also strongly correlated with permethrin and cypermethrin (Fig. 2).

Inter-matrix correlations were modest. For p,p′-DDE, coefficients were 0.24 (CSF—hair) and 0.57 (serum—hair). Between serum and CSF, correlations were 0.38 for p,p′-DDE and 0.27 for β-HCH (Figure S1). In the subset of 14 infants with all three matrices, p,p′-DDE was the sole compound detected in all samples, showing a moderate correlation between hair and serum (0.55), and weak correlations between CSF and hair (0.15) and between CSF and serum (−0.15) (Figure S1).

### Factorial and clustering analyses

In hair, the first and second MCA dimensions explained 20.9% and 11.2% of the variance, driven primarily by pyrethroids (permethrin, *cis*-Cl2CA, trans-Cl2CA) and fipronil sulfone. Four clusters were identified, differing by pesticide family, particularly Cluster 2 (high p,p′-DDE detection) and Cluster 4 (high pyrethroid detection) (Table S1). Hair-based clusters from hair analysis were not associated with any cofactors (Table S2).

In serum, the first and second dimensions explained 31.7% and 14.6% of the variance, driven by HCB, p,p′-DDT, and p,p′-DDE. Three clusters corresponded to low (Cluster 1), medium (Cluster 2), and high (Cluster 3) detection levels (Table S3). Breastfeeding was strongly associated with clustering, with an OR of 4.46 (1.29;20.9) for Cluster 2 and 20.3 (4.70;117) for Cluster 3, compared with Cluster 1. Birthweight was negatively associated for Cluster 2 (OR 0.1, 0.03;0.37) (Table S4).

In CSF, owing to low detection rates (only β-HCH and p,p′-DDE > 10%), the first MCA dimension explained 85.3% of the variance. Two clusters were identified: Cluster 1 (82.5% of subjects, no detections) and Cluster 2 (majority of detections) (Table S5). Age and breastfeeding were associated with clustering; younger age (OR 6.04, 1.93;23) and breastfeeding (OR 7.04, 1.99;33.3) were both associated with Cluster 2 (Table S6).

### Sensitivity analyses

Re-running MCA and HCPC with uniform classification profiles (two classes) showed stable results. Linear regression using continuous data (age and birthweight) supported the categorical analysis, with a higher birthweight in Cluster 2 and 3 in serum, and a lower age in Cluster 2 for CSF (Table S7). Although the age data were not normally distributed, we retained linear regression, as the aim was exploratory to assess consistency with the categorical analysis.

## Discussion

This study is essentially descriptive. It is not an etiological study on SUDI, and we cannot estimate the association between pesticide exposure and SUDI, or any other infant outcomes. The cluster-based exposure analysis and its association with other factors also remain exploratory.

Nevertheless, we detected a wide range of pesticides, including persistent organochlorines (OCs), pyrethroids (PYRs), and phenylpyrazoles (PAs), in postmortem samples from SUDI cases. The detection of OCs is particularly concerning given their established toxicity and persistence. Although OCs are banned, their ubiquity in serum and hair reflects long-term exposure and environmental persistence. The presence of PYRs and PAs in hair underscores ongoing domestic and veterinary exposure pathways.

Comparison with the existing literature yields mixed but informative results. Our serum OC detection rates align closely with the French PELAGIE cohort [22], particularly for β-HCH, HCB and p,p′-DDE. However, our detection rates were lower than those reported in cord blood in China [23]. In hair, our detection of lindane (91.2%) contrasts sharply with studies in Chinese newborns (0%) [24], but aligns with a French cohort of older children (100%) [25]. One study analyzed neonicotinoid pesticides in CSF samples from children aged three to 18 years with leukemia or lymphoma in Switzerland and detected acetamiprid in 13 of the 14 CSF samples [26]. By contrast, our study focused on OCs.

Overall, our detection rates were low, particularly in hair, consistent with similar studies [23, 24, 26]. Because detection rates depend on LOD, which varied across studies, direct quantitative comparisons should be interpreted with caution.

Our findings reveal strong intra-matrix correlations among OC markers in serum and CSF, and among specific metabolites in hair, suggesting a range of exposure profiles, from low to high levels of exposure to multiple compounds. Consistent with known metabolic pathways, strong correlations were observed between parent compounds and metabolites (e.g., permethrin/cis-Cl2CA), between closely related compounds (p,p′-DDE/p,p′-DDT), and between co-applied compounds (BPO/pyrethroids – BPO acts as a synergist of pyrethroids). Strong correlations among OCs likely point to a common exposure source, whereas weak correlations between different pesticide families in hair (e.g., organophosphates vs. pyrethroids) more likely reflect diverse exposure origins, such as agricultural crops versus domestic insecticides or veterinary treatments.

However, the lack of correlation for p,p′-DDE between CSF and other matrices suggests limited transfer or rapid clearance in the central nervous system, or possibly matrix-specific detection biases.

Correlations between matrices are difficult to interpret, owing to the limited number of pesticides common to the different matrices and the small number of infants for whom data were available across all three matrices. Nonetheless, these matrices likely reflect different windows of exposure (hair, for example, may capture cumulative exposure over several months) and may differ in their ability to retain lipophilic compounds [27, 28].

Exposure clusters were significantly associated with infant characteristics: breastfeeding and higher birthweight were linked to higher OC detection in serum, while breastfeeding and younger age (0–2 months) were associated with higher detection in CSF. This last result requires further validation to assess potentially different mechanisms during the first weeks of life, along with related hypotheses such as greater blood–brain barrier permeability.

In serum and CSF, we observed an association between breastfeeding and high levels of OC detection, likely explained by the ability of OCs to accumulate in breast milk and subsequently transfer to the infant [29], despite the overall positive impact of breastfeeding on children's health [30]. However, we were unable to determine the type of breastfeeding (mixed or exclusive) or its duration.

While pesticide exposure has been associated with preterm birth and low birthweight in previous studies [5], we observed no clear associations between these factors and clusters with high levels of exposure. However, our population consisted exclusively of dead infants, introducing selection bias that does not reflect the general population of infants.

This study represents the first assessment of pesticide exposure in French SUDI cases, and one of the largest international studies to include biological samples from three matrices, including CSF. We analyzed both current and banned neurotoxic pesticides and identified associations with key cofactors such as age and breastfeeding.

However, several limitations must be acknowledged. The primary constraint was the small sample volumes, particularly for hair and CSF, which resulted in variable and potentially high LODs. This likely reduced detection rates and obscured true exposure levels, limiting comparisons with studies using lower LODs. Despite the complex nature of pesticide exposure, we analyzed only OCs in serum and CSF samples. Furthermore, while clustering is a powerful tool for identifying exposure patterns, the results are sensitive to data type, cluster number, and detection frequency. However, our sensitivity analyses confirmed two associations: age in CSF and breastfeeding in serum/CSF, providing a solid foundation for future research. Cluster-based analysis of hair samples was limited by the extensive missing data, particularly for birthweight, breastfeeding, sleep position, preterm birth, and social deprivation. We performed a large number of tests and analyses, which increases the probability of finding associations by chance. However, we chose not to correct the data to avoid masking subtle effects, as part of an exploratory approach.

Future studies should adopt a comparative case–control design to assess the role of pesticides in SUDI. Control groups could include living infants or infants who died from causes unrelated to exposure (e.g., trauma). Hair sample collections are particularly valuable for such designs owing to their non-invasive nature and their ability to capture long-term exposure. Our data can help determine the required sample size and prioritize specific pesticides for investigation.

CSF analysis remains a promising approach for identifying neurotoxic substances that have crossed the blood–brain barrier, particularly given the neurotoxic hypotheses surrounding SUDI. Future studies should focus on expanding the panel of detectable compounds in this matrix, including neonicotinoids [26] and other neurotoxic pollutants such as heavy metals, PFAS, PCBs, and solvents. Additionally, CSF samples from infants with negative lumbar punctures could serve as valuable controls in etiological studies.

This study confirms the presence of diverse pesticides, including persistent organochlorines banned decades ago, in serum, hair, and CSF from SUDI cases. While the exploratory design does not permit causal inference between exposure and SUDI, we identified distinct exposure clusters associated with infant age or breastfeeding status in serum and CSF. These findings underscore pesticide exposure in early life and the need for further studies on their effects on infants’ health. The analysis of CSF and hair matrices collected during post-mortem examinations should be considered in future investigations in this population. Future research should prioritize larger case–control studies with optimized sensitivity to validate these associations, assess the potential role of pesticide exposure in SUDI, and identify maternal exposures.

## Supplementary information

Below is the link to the electronic supplementary material.

ESM 1 (DOCX 809 KB)

## Data Availability

No datasets were generated or analysed during the current study.

## Abbreviations

SUDI: Sudden unexpected death in infancy
MCA: Multiple correspondence analysis
HCPC: Hierarchical clustering on principal components
CSF: Cerebrospinal fluid
OC: Organochlorine
OP: Organophosphate
PYR: Pyrethroid
PA: Phenylpyrazole
CA: Carbamate
A-PYR: Pyrethroid adjuvants
NN: Neonicotinoid
DDD: Dichlorodiphenyldichloroethane
DDE: Dichlorodiphenyldichloroethylene
DDT: Dichlorodiphenyltrichloroethane
HCH: Hexachlorocyclohexane
HCB: Hexachlorobenzene
PeCBz: Pentachlorobenzene
*cis*-Br2CA: *cis*-3-(2,2Dibromovinyl)-2,2-dimethylcyclopropane-carboxylic acid
*cis*-ClCF3CA: 3-(2-Chloro-3,3,3-trifluoro-1-propenyl)-2,2-dimethylcyclopropanecarboxylic acid
IMPH: 2‑Isopropyl‑4‑methyl‑6‑hydroxypyrimidine
Cl2CA: *cis*-3-(2,2Dichlorovinyl)-2,2-dimethylcyclopropane-carboxylic acid
BPO: Benzoyl peroxide
TCPy: 3,5,6-Trichloro-2-pyridinol
