# Supplementary material for: Pesticide detection in postmortem serum, hair, and cerebrospinal fluid from cases of sudden unexpected death in infancy: a registry-based study
Source: Eur J Pediatr. 2026 Oct 24;185(11):886. doi: 10.1007/s00431-026-07546-6 (PMC13695688; doi:10.1007/s00431-026-07546-6)
Supplement: Supplementary file 1 [file 431_2026_7546_MOESM1_ESM.docx]

**Supplementary material**

**Pesticide detection in postmortem serum, hair, and cerebrospinal fluid from cases of sudden unexpected death in infancy: a registry-based study**

**Table of contents**

[Sampling methods	3](#_Toc242259198)

[Cofactors and logistic regression methods	3](#_Toc242259199)

[Hair analysis method	4](#_Toc242259200)

[Serum and CSF analysis methods	9](#_Toc242259201)

[Figure S1: Polychoric correlations between matrices, for pesticides detected in more than 10% of samples	11](#_Toc242259202)

[Figure S2: Results of MCA and HCPC analyses on hair samples (n=57)	12](#_Toc242259203)

[Figure S3: MCA and HCPC analyses on serum samples (n=98)	13](#_Toc242259204)

[Figure S4: MCA and HCPC analyses on CSF samples (n=97)	14](#_Toc242259205)

[Table S1: Distribution of exposures and cofactors in hair clusters (n=57). Clustering was performed based on the MCA for exposure data	15](#_Toc242259206)

[Table S2: Results from logistic regression between cluster-based exposure and factors, in hair (n=57)	16](#_Toc242259207)

[Table S3: Distribution of exposures in serum clusters (n=98)	17](#_Toc242259208)

[Table S4: Results from logistic regression between cluster-based exposure and factors, in serum (n=98)	18](#_Toc242259209)

[Table S5: Distribution of exposures in CSF clusters (n=97). Clustering was performed based on the MCA for exposure data	19](#_Toc242259210)

[Table S6: Results from logistic regression between cluster-based exposure and factors, in CSF (n=97)	19](#_Toc242259211)

[Table S7: Results from linear regression between cluster-based exposure and continuous factors	20](#_Toc242259212)

[Example of R code	21](#_Toc242259213)

[References	23](#_Toc242259214)

# Sampling methods

In France, the protocol regarding SUDI is developed by the SUDI registry “OMIN”. When a SUDI occurred in France, the regional SUDI center received the parents and the infant body. There are 35 regional centers in 2026 – including four centers in overseas territories – with several health workers (nurses, MD, etc.) (<https://www.omin.fr/centres-participants/>). Following the national protocol, they perform data and sample collection. Regarding particularly the biological samples, collected samples are stored in the regional center, with a -80°C frozen immediately after collection for serum and CSF, and packaging at room temperature for hair. Once to twice per year, all data and samples are transferred to the registry, in Nantes Hospital. Samples are stored in Nantes University Hospital (CHU Nantes, Hôtel Dieu, Centre de Ressources Biologiques (CRB), Nantes, F44093, France (BRIF: BB-0033-00040)). (<https://www.omin.fr/>)

# Cofactors and logistic regression methods

We included several cofactors in the analysis to assess their potential association with clusters. Age at death was dichotomized by 0-2 months and ≥3 months, following precedent studies on SUDI (1,2). Birthweight was dichotomized by <2500g and ≥2500g, as the first category represent the common definition of low birthweight (3). Because of our limited number of data and our approach remain exploratory; we did not include several categories of preterm birth. We conducted a logistic regression for each categorical factor, comparing the different clusters with cluster 1.

# Hair analysis method

Hair sample preparation including washing and grinding

The hair sample was first removed from its sealed paper envelope, measured using a metal ruler and then weighed on a sheet of paper. When necessary, the sample was cut using ceramic scissors. For this study, it was decided to work on the entire hair sample because, i) the available mass for many samples was too small to consider analyzing only part of the sample, ii) the root was difficult to identify for most samples and iii) the hair was generally ‘loose’ in the envelope. The washing step is intended to remove external contaminants and potential interfering substances that may have been deposited or adsorbed onto the hair surface. However, it was decided not to wash the hair sample before analysis for several reasons, the main one being that the effectiveness of this step is very difficult to control and can potentially vary from one chemical family to another, or even from one molecule to another. Other concerns include possible removal of hair incorporated compounds, risks of contamination or loss during sample processing, and lower detection frequencies due to the reduction in concentrations caused by washing. The hair sample was thus pulverized directly using a Retsch MM400 mixer mill (Retsch GmbH, Haan, Germany) (10 minutes at 25 Hz). The resulting powder obtained was collected on a piece of paper and transferred into an amber glass vial previously rinsed with dichloromethane. Target compounds were extracted using acetonitrile. To do this, 20 mg of hair powder were weighed into an Eppendorf tube, followed by the addition of 500 µL of acetonitrile and the internal standards (ISTDs). The Eppendorf tube was vortexed before being placed in an Edmund Bühler TH 15 shaker/incubator (Edmund Bühler GmbH, Bodelshausen, Germany) at room temperature for 2 hours and then centrifuged at 20°C at 8,000 rpm for 20 minutes using a Fisher Scientific accuSpin Micro 17R microcentrifuge (Fisher Scientific S.A.S., Illkirch, France). After centrifugation, the supernatant was transferred to a second Eppendorf tube. 500 µL of acetonitrile were added again to the first tube containing the remaining hair powder, and the extraction procedure was repeated, excluding the shaking/incubation step. The second supernatant was combined to the first, and the mixture underwent a final centrifugation step at 20°C at 8,000 rpm for 5 minutes to remove residual hair particles. The resulting organic extract was transferred to a third Eppendorf tube and concentrated to dryness in a water bath at 30°C under a nitrogen stream using a N-EVAP 111 Organomation Nitrogen Evaporator (Organomation, Berlin, MA, USA). The residue was then reconstituted in 200 µL of dichloromethane, vortexed and briefly sonicated. The resulting extract was divided into two 1.5 mL amber glass vials fitted with a 250 µL insert (100 µL in each vial), which were then sealed with an aluminum cap and finally stored in a freezer (-20°C) until analysis by gas chromatography coupled with tandem mass spectrometry (GC/MS/MS).

GC/MS/MS analysis

The first vial was analyzed directly, while the second vial was analyzed after a derivatization step by adding 15 µL of N-methyl-N-trimethylsilyl-trifluoroacetamide (MSTFA). Analyses were performed using a 7890B GC system coupled to a 7010B GC/MS Triple Quad (Agilent Technologies, Santa Clara, California, United States) operated in electron impact ionization (EI) mode (70 eV). The GC system was equipped with a 7693 Autosampler and a multimode inlet (MMI) fitted with an empty (no wool) single taper ultra-inert glass liner. Calibration solutions and sample extracts were injected (2 µL) in the solvent vent mode (vent pressure: 0 psi until 0.1 min; purge flow to split vent: 60 mL/min at 1.5 min; vent flow: 100 mL/min) with the following injector temperature program: 37 °C (hold 0.1 min), ramp at 500 °C/min to 325 °C (hold 10 min). Helium was used as the column carrier gas at a constant flow rate of 1.0 mL/min. Chromatographic separation was performed on a Rtx®-PCB capillary column (30 m length × 0.25 mm I.D., 0.25 µm film thickness) supplied by Restek Corporation (Bellefonte, Pennsylvania, United States) with the following oven temperature program: 37 °C (hold 2 min), first ramp at 37 °C/min to 250 °C (hold 0 min), second ramp at 15 °C/min to 320 °C (hold 8 min to reach an analysis time of 20.4 min). The MSD transfer line, ion source and quadrupole temperatures were fixed at 325, 280 and 150 °C, respectively. The mass spectrometer (triple quadrupole) was operated in multiple reaction monitoring (MRM) mode. The two most intense and specific MRM transitions of each compound (quantifier and qualifier transitions) were monitored for identification, confirmation and quantification. They were selected using the pesticides and environmental pollutants MRM database provided by Agilent Technologies for the compounds present in the database or following the usual procedure for others. MassHunter software (B.07.06) was used for instrument control, data acquisition and quantification. The samples were analyzed in March and April 2025.

Quality assurance and quality control (QA/QC)

The limit of detection (LOD) was defined as the lowest concentration of a substance that can be distinguished from the absence of that substance. LODs were estimated from the replicate analysis of a blank sample. The limit of quantification (LOQ) was defined as the lowest concentration of a substance for which the relative standard deviation (RSD) of the raw signal (n = 5) was lower than or equal to 20%, the signal-to-noise ratio (S/N) was greater than or equal to 10, and the raw signal was greater than or equal to 5 times the signal of the blank sample.

Seventeen labeled and non-labeled substances were selected to best cover the physical and chemical properties of the targeted analytes. 4,4'-DDE D8, acetamiprid D3, BPO D9, DBCA 13C2, diazinon D10, fenpropathrin, fipronil desulfinyl 15N2 13C4, fipronil sulfone 15N2 13C4, fosthiazate D5, HCB 13C6, IMPH 13C4, phorate diethoxy 13C4, TCPy 13C3, trans-cypermethrin D6, trans-DCCA 13C2, β-HCH 13C6 and γ-HCH 13C6 were added prior to the extraction step and used as internal standards (ISTDs). All compounds were quantified using the appropriate ISTD to compensate for the variability associated with the analytical procedure, from calibration curves generated for each compound by analyzing at least four different calibration samples. A quadratic fit (origin ignored, no weighting) was used to compensate for the nonlinearity of the instrument response over a wide working range.

Each batch included: i) up to 18 hair samples (20 mg), ii) one calibration blank sample and six calibration samples to generate quadratic calibration curves intended for quantification, iii) several calibration samples prepared from commercial solutions provided by other suppliers and analyzed as regular samples to validate the preparation of the calibration solutions, iv) several calibration samples analyzed at the beginning, throughout and at the end of the batch to check for the stability of the detector response, v) one procedural blank sample analyzed as regular samples to assess whether contamination may have occurred during analysis, vi) two procedural QC samples, spiked at the LOQ level and at an intermediate level, analyzed as regular samples to check for method accuracy and for the stability of the detector response, and vii) three matrix procedural QC samples (20-mg real hair sample non-spiked and spiked at the LOQ level and at an intermediate level) analyzed as regular samples to check for method accuracy and for the stability of the detector response.

Positive values for each substance were confirmed by comparing retention times and MRM transitions ratios (±30% for a concentration greater than or equal to the LOQ level) between calibration and hair samples. The data validation protocol included several conditions: i) the determination coefficient of the calibration curve had to be greater than 0.99, ii) the concentration of a substance measured in the calibration samples had to be within ±50% of its theoretical concentration value at the LOD and LOQ levels and ±25% at all other levels, iii) the response of a substance (ISTD response ratio) in the calibration and procedural blank samples had to be lower than 50% of that in the calibration sample at the LOQ level, iv) the concentration of a substance measured in the calibration samples prepared from commercial solutions provided by other suppliers had to be within ±25% of its theoretical concentration value, v) the concentration of a substance measured in the calibration samples analyzed at the beginning, throughout and at the end of the batch had to be within ±25% of its theoretical concentration value, vi) the concentration of a substance measured in the procedural QC samples had to be within ±50% of its theoretical concentration value at the LOQ level and ±30% at the intermediate level, vii) the concentration of a substance measured in the matrix procedural QC samples prepared from a real hair sample had to be within ±50% of its theoretical concentration value at the LOQ level and ±30% at the intermediate level (after subtracting the concentration of the same substance measured in the non-spiked matrix procedural QC sample), and viii) the concentration of a substance measured in the hair samples had to be within the method working range without exceeding 110% of the concentration of the most concentrated calibration sample. If all these conditions were not met, the results were not validated and the samples in question were reanalyzed whenever possible.

# Serum and CSF analysis methods

Serum and CSF samples were analyzed by LABERCA in Nantes, France. The samples analyzed (approximately 1 g for serum samples and 0.2 - 0.5 g for CSF samples), blanks and QC samples (1 mL), were mixed with formic acid (in proportions equivalent to those for the test samples). C18 cartridges were conditioned with nHex, EtOH, MeOH and H2O. The internal standard working solution was loaded onto each cartridge prior to the hydrolyzed samples were diluted with H2O (in proportions equivalent to those for the test samples). The cartridges were rinsed with H2O and dried under vacuum with a carbon cartridge on top of each C18 cartridge. Elution was performed with nHexane. The eluate was concentrated down to approximately 200 µL. Florisil cartridges were conditioned with diethyl ether/DCM (80:20) before the loading of 200 µL of concentrate. Elution was performed with diethyl ether/DCM (80:20). The eluate was concentrated down and transferred into insert vials. Recovery standard working solution was added to each vial before injection. Analyses were performed on a 7890 (Agilent Technologies) gas chromatograph equipped with a 7693 (Agilent Technologies) autosampler coupled to a 7010 mass spectrometer (Agilent Technologies). Injections (2 μL) were performed in a split/splitless liner (Agilent Technologies) held at 250 °C in splitless mode. Separation was performed on an OPTIMA 17MS column (30 m × 0.25 mm, 0.15 µm; Macherey-Nagel). The carrier gas was helium at a flow rate of 1 mL/min. The temperature gradient was as follows: 120 °C, increased by 20 °C/min to 180 °C, then increased at 5 °C/min to 290 °C, and finally increased at 20 °C/min to a final temperature of 300 °C. Quantification was performed according to isotope dilution methods with labeled internal standards.

An analytical run consisted of 20 samples, 2 blanks (UP water) and 1 quality control (doped serum). The method has been fully validated according to the procedures in force in our laboratory, and has been submitted to several international inter-laboratory trials (G-EQUAS & AMAP), which confirmed its robustness and accuracy.

LODs and LOQs were defined according to the following principles:

- Where the analyte is not detected in the blanks, the LOD is set at the instrumental limit, i.e. the lowest point at which the signal yields a signal-to-noise ratio of 3
- Where the analyte is quantified in the blanks, the LOD is set at three times the mean value of the two blanks; for these analytes, the LOD varies from one series to another
- The LOQ corresponds, by definition, to the lowest calibration point considered valid according to our specifications (relative response factor below 20%). When the target compound is present in the blanks, the LOQ is instead derived from the LOD as LOQ = 3 × LOD
- Furthermore, the LOD is expressed relative to the sample volume of each sample, which can vary significantly – particularly for CSF, where some samples were received with volumes two or even four times greater than others

a. Serum and CSF (n=31)	b. CSF and hair (n=12)

c. Serum and hair (n=13)	d. CSF, hair and serum (n=14)

Figure S1: Polychoric correlations between matrices, for pesticides detected in more than 10% of samples (a: serum and CSF, b: CSF and hair, c: serum and hair, d: CSF, hair and serum)

*Abbreviations: CSF, cerebrospinal fluid; DDE, dichlorodiphenyldichloroethylene; HCH, hexachlorocyclohexane. The color key ranges from red for very strong positive correlations to blue for strong negative correlations. Pink and pale blue indicate moderate positive and negative correlations, respectively. Blank indicates no correlation.*

a. MCA results

b. HCPC results

Figure S2: Results of MCA and HCPC analyses on hair samples (n=57) (a: MCA results, b: HCPC results)

*Abbreviations: DDE, dichlorodiphenyldichloroethylene; MCA, Multiple correspondence analysis; HCPC, hierarchical clustering on principal components.*

a. MCA results

b. HCPC results

Figure S3: MCA and HCPC analyses on serum samples (n=98) (a: MCA results, b: HCPC results)

*Abbreviations: DDE, dichlorodiphenyldichloroethylene; DDD, dichlorodiphenyldichloroethane; DDT, dichlorodiphenyltrichloroethane; HCH, hexachlorocyclohexane; HCB, hexachlorobenzene; MCA, multiple correspondence analysis; HCPC, hierarchical clustering on principal components.*

a. MCA results

b. HCPC results

Figure S4: MCA and HCPC analyses on CSF samples (n=97) (a: MCA results, b: HCPC results)

*Abbreviations: CSF, cerebrospinal fluid; DDE, dichlorodiphenyldichloroethylene; HCH, hexachlorocyclohexane; MCA, multiple correspondence analysis; HCPC, hierarchical clustering on principal components.*

# Table S1: Distribution of exposures and cofactors in hair clusters (n=57). Clustering was performed based on the MCA for exposure data

| **Variable** | **Category** | **Cluster (n)** | | | | *p*-value |
| --- | --- | --- | --- | --- | --- | --- |
|  |  | **1 (24)** | **2 (14)** | **3 (8)** | **4 (11)** |  |
| *Cis*-Cl2CA (%) | *Cis*-Cl2CA_1 | 24 (100.0) | 14 (100.0) | 5 (62.5) | 0 (0.0) | <0.001 |
|  | Cis-Cl2CA_2 | 0 (0.0) | 0 (0.0) | 3 (37.5) | 11 (100.0) |  |
| *Trans*-Cl2CA (%) | *Trans*-Cl2CA_1 | 22 (91.7) | 12 (85.7) | 3 (37.5) | 0 (0.0) | <0.001 |
|  | Trans-Cl2CA_2 | 2 (8.3) | 2 (14.3) | 5 (62.5) | 11 (100.0) |  |
| Fipronil desulfinyl (%) | Fipronil desulfinyl_1 | 23 (95.8) | 14 (100.0) | 0 (0.0) | 11 (100.0) | <0.001 |
|  | Fipronil desulfinyl_2 | 1 (4.2) | 0 (0.0) | 8 (100.0) | 0 (0.0) |  |
| p,p'-DDE (%) | p,p'-DDE_1 | 21 (87.5) | 3 (21.4) | 4 (50.0) | 5 (45.5) | 0.001 |
|  | p,p'-DDE_2 | 3 (12.5) | 11 (78.6) | 4 (50.0) | 6 (54.5) |  |
| Propoxur (%) | Propoxur_1 | 22 (91.7) | 10 (71.4) | 7 (87.5) | 11 (100.0) | 0.149 |
|  | Propoxur_2 | 2 (8.3) | 4 (28.6) | 1 (12.5) | 0 (0.0) |  |
| Transfluthrin (%) | Transfluthrin_1 | 22 (91.7) | 7 (50.0) | 2 (25.0) | 8 (72.7) | 0.002 |
|  | Transfluthrin_2 | 2 (8.3) | 7 (50.0) | 6 (75.0) | 3 (27.3) |  |
| Permethrin (%) | Permethrin_1 | 24 (100.0) | 8 (57.1) | 2 (25.0) | 2 (18.2) | <0.001 |
|  | Permethrin_2 | 0 (0.0) | 6 (42.9) | 6 (75.0) | 9 (81.8) |  |
| BPO (%) | BPO_1 | 22 (91.7) | 12 (85.7) | 3 (37.5) | 4 (36.4) | 0.001 |
|  | BPO_2 | 2 (8.3) | 2 (14.3) | 5 (62.5) | 7 (63.6) |  |
| TCPy (%) | TCPy_1 | 10 (41.7) | 8 (57.1) | 4 (50.0) | 3 (27.3) | 0.764 |
|  | TCPy_2 | 8 (33.3) | 3 (21.4) | 2 (25.0) | 3 (27.3) |  |
|  | TCPy_3 | 6 (25.0) | 3 (21.4) | 2 (25.0) | 5 (45.5) |  |
| Fipronil sulfone (%) | Fipronil sulfone_1 | 16 (66.7) | 3 (21.4) | 0 (0.0) | 5 (45.5) | <0.001 |
|  | Fipronil sulfone_2 | 6 (25.0) | 7 (50.0) | 0 (0.0) | 4 (36.4) |  |
|  | Fipronil sulfone_3 | 2 (8.3) | 4 (28.6) | 8 (100.0) | 2 (18.2) |  |
| Cypermethrin (%) | Cypermethrin_1 | 16 (66.7) | 4 (28.6) | 3 (37.5) | 2 (18.2) | 0.001 |
|  | Cypermethrin_2 | 5 (20.8) | 8 (57.1) | 2 (25.0) | 1 (9.1) |  |
|  | Cypermethrin_3 | 3 (12.5) | 2 (14.3) | 3 (37.5) | 8 (72.7) |  |
| Lindane (%) | Lindane_1 | 12 (50.0) | 1 (7.1) | 1 (12.5) | 1 (9.1) | 0.003 |
|  | Lindane_2 | 9 (37.5) | 2 (14.3) | 1 (12.5) | 4 (36.4) |  |
|  | Lindane_3 | 3 (12.5) | 4 (28.6) | 2 (25.0) | 3 (27.3) |  |
|  | Lindane_4 | 0 (0.0) | 7 (50.0) | 4 (50.0) | 3 (27.3) |  |

*Clustering was performed based on the MCA for exposure data*. *P-values were obtained by exact Fischer test. Abbreviations: DDE, dichlorodiphenyldichloroethylene; MCA, multiple correspondence analysis. For exposure categories, classes are defined on the basis of increasing detection rates (i.e. “_2” corresponds to higher detection rates than “_1”).*

# Table S2: Results from logistic regression between cluster-based exposure and factors, in hair (n=57)

|  | **Age**  **(n=57)** | **Birthweight**  **(n=34)** | **Breastfeeding**  **(n=35)** | **Position**  **(n=39)** | **Sex**  **(n=57)** | **Preterm birth**  **(n=35)** | **Fdep**  **(n=19)** |
| --- | --- | --- | --- | --- | --- | --- | --- |
| Cluster 1 (n=24) | Ref | Ref | Ref | Ref | Ref | Ref | Ref |
| Cluster 2 (n=14)  *OR (95%CI)* | 0.4  (0.11;1.86 |  | 2.80  (0.48;19.1) | 0.54  (0.03;4.73) | 1.00  (0.26;3.79) | 1.71  (0.17;17.1) | 1.49  (0.30;7.60) |
| Cluster 3  (n=8)  *OR (95%CI)* | 1.18  (0.23;6.11) |  | 2.33  (0.38;16.3) | 2.33  (0.26;17.6) | 0.33  (0.04;1.79) |  | 0.80  (0.18;3.51) |
| Cluster 4  (n=11)  *OR (95%CI)* |  |  | 1.40  (0.19;10.7) |  | 1.75  (0.41;8.20) |  | 5.94  (0.97;51.0) |

*Abbreviations:* *Fdep, French deprivation index. The gray areas indicate a lack of data. The “n” at the top of each column indicates the number of data used after removing the unknown data.*

# Table S3: Distribution of exposures in serum clusters (n=98)

| **Variable** | **Category** | **Cluster (n)** | | | ***p*-value** |
| --- | --- | --- | --- | --- | --- |
|  |  | **1 (26)** | **2 (50)** | **3 (22)** |  |
| *Trans*-nonachlor (%) | *Trans*-nonachlor_1 | 26 (100.0) | 46 (92.0) | 2 (9.1) | <0.001 |
|  | *Trans*-nonachlor_2 | 0 (0.0) | 4 (8.0) | 20 (90.9) |  |
| Dieldrin (%) | Dieldrin_1 | 26 (100.0) | 35 (70.0) | 4 (18.2) | <0.001 |
|  | Dieldrin_2 | 0 (0.0) | 15 (30.0) | 18 (81.8) |  |
| p,p'-DDD (%) | p,p'-DDD_1 | 25 (96.2) | 42 (84.0) | 7 (31.8) | <0.001 |
|  | p,p'-DDD_2 | 1 (3.8) | 8 (16.0) | 15 (68.2) |  |
| β-HCH (%) | β-HCH_1 | 21 (80.8) | 8 (16.0) | 0 (0.0) | <0.001 |
|  | β-HCH_2 | 3 (11.5) | 28 (56.0) | 4 (18.2) |  |
|  | β-HCH_3 | 2 (7.7) | 14 (28.0) | 18 (81.8) |  |
| *Cis*-heptachlor epoxide (%) | *Cis*-heptachlor epoxide_1 | 22 (84.6) | 9 (18.0) | 0 (0.0) | <0.001 |
|  | *Cis*-heptachlor epoxide_2 | 4 (15.4) | 28 (56.0) | 2 (9.1) |  |
|  | *Cis*-heptachlor epoxide_3 | 0 (0.0) | 13 (26.0) | 20 (90.9) |  |
| p,p'-DDT (%) | p,p'-DDT_1 | 25 (96.2) | 23 (46.0) | 0 (0.0) | <0.001 |
|  | p,p'-DDT_2 | 1 (3.8) | 21 (42.0) | 3 (13.6) |  |
|  | p,p'-DDT_3 | 0 (0.0) | 6 (12.0) | 19 (86.4) |  |
| p,p'-DDE (%) | p,p'-DDE_1 | 20 (76.9) | 5 (10.0) | 0 (0.0) | <0.001 |
|  | p,p'-DDE_2 | 3 (11.5) | 21 (42.0) | 0 (0.0) |  |
|  | p,p'-DDE_3 | 2 (7.7) | 17 (34.0) | 5 (22.7) |  |
|  | p,p'-DDE_4 | 1 (3.8) | 7 (14.0) | 17 (77.3) |  |
| HCB (%) | HCB_1 | 24 (92.3) | 4 (8.0) | 0 (0.0) | <0.001 |
|  | HCB_2 | 1 (3.8) | 22 (44.0) | 1 (4.5) |  |
|  | HCB_3 | 1 (3.8) | 19 (38.0) | 3 (13.6) |  |
|  | HCB_4 | 0 (0.0) | 5 (10.0) | 18 (81.8) |  |

*Clustering was performed based on the MCA for exposure data*. *P-values were obtained by exact Fischer test. Abbreviations: DDE, dichlorodiphenyldichloroethylene; DDT, dichlorodiphenyltrichloroethane; HCH, hexachlorocyclohexane; HCB, hexachlorobenzene; MCA, multiple correspondence analysis. For exposure categories, classes are defined according to increasing detection rates (i.e. “_2” corresponds to higher detection rates than “_1”).*

# Table S4: Results from logistic regression between cluster-based exposure and factors, in serum (n=98)

|  | **Age**  **(n=98)** | **Birthweight**  **(n=89)** | **Breastfeeding**  **(n=89)** | **Position**  **(n=98)** | **Sex**  **(n=98)** | **Preterm birth**  **(n=91)** | **Fdep**  **(n=94)** |
| --- | --- | --- | --- | --- | --- | --- | --- |
| Cluster 1  (n=26) | Ref | Ref | Ref | Ref | Ref | Ref | Ref |
| Cluster 2  (n=50)  *OR (95%CI)* | 0.88  (0.31;2.54) | 0.1  (0.03;0.37) | 4.46  (1.29;20.9) | 1.25  (0.31;6.21) | 1.40  (0.53;3.67) | 0.27  (0.08;0.86) | 1.53  (0.65;3.66) |
| Cluster 3  (n=22)  *OR (95%CI)* | 1.05  (0.3;3.6) |  | 20.3  (4.70;117) |  | 0.86  (0.27;2.68) | 0.17  (0.02;0.8) | 0.77  (0.28;2.1) |

*Abbreviations:* *Fdep, French deprivation index. The gray areas indicate a lack of data. The “n” at the top of each column indicates the number of data used after removing the unknown data.*

# Table S5: Distribution of exposures in CSF clusters (n=97). Clustering was performed based on the MCA for exposure data

| **Variable** | **Category** | **Cluster (*n*)** | | *p*-value |
| --- | --- | --- | --- | --- |
|  |  | **1 (80)** | **2 (17)** |  |
| β-HCH (%) | β-HCH_1 | 80 (100.0) | 5 (29.4) | <0.001 |
|  | β-HCH_2 | 0 (0.0) | 12 (70.6) |  |
| p.p..DDE (%) | p.p..DDE_1 | 80 (100.0) | 2 (11.8) | <0.001 |
|  | p.p..DDE_2 | 0 (0.0) | 15 (88.2) |  |

*Clustering was performed based on the MCA for exposure data*. *P-value obtained by exact Fischer test. Abbreviations: DDE, dichlorodiphenyldichloroethylene; HCH, hexachlorocyclohexane; MCA, multiple correspondence analysis. For exposure categories, classes are defined according to increasing detection rates (i.e. “_2” corresponds to higher detection rates than “_1”).*

# Table S6: Results from logistic regression between cluster-based exposure and factors, in CSF (n=97)

|  | **Age**  **(n=97)** | **Birthweight**  **(n=91)** | **Breastfeeding**  **(n=87)** | **Position**  **(n=97)** | **Sex**  **(n=97)** | **Preterm birth**  **(n=92)** | **Fdep**  **(n=92)** |
| --- | --- | --- | --- | --- | --- | --- | --- |
| Cluster 1  (n=80) | Ref | Ref | Ref | Ref | Ref | Ref | Ref |
| Cluster 2  (n=17)  *OR (95%CI)* | 6.04  (1.93;23) | 0.95  (0.24;3.14 | 7.04  (1.99;33.3) | 0.27  (0.01;1.50) | 2.17  (0.73;7.35) | 1.07  (0.27;3.53) | 1.24  (0.49;3.12) |

*Abbreviations:* *Fdep, French deprivation index. The “n” at the top of each column indicates the number of data used after removing the unknown data.*

# Table S7: Results from linear regression between cluster-based exposure and continuous factors

|  |  | **Age (months)** | **Birthweight (g)** |
| --- | --- | --- | --- |
| Hair  (n=57) | Cluster 1 | Ref | Ref |
|  | Cluster 2  *ß (95%CI)* | -0.54 (-5.3;4.3) | 6.7 (-30;333) |
|  | Cluster 3  *ß (95%CI)* | 1.2 (-4.6;7.0) | -38 (-434;358) |
|  | Cluster 4  *ß (95%CI)* | 6.3 (1.1;12) | 20 (-333;374) |
| Serum  (n=98) | Cluster 1 | Ref | Ref |
|  | Cluster 2  *ß (95%CI)* | 2.3 (-0.27;4.8) | 551 (271;831) |
|  | Cluster 3  *ß (95%CI)* | -0.20 (-3.3;2.8) | 768 (432;1103) |
| CSF | Cluster 1 | Ref | Ref |
| (n=97) |  |  |  |
|  | Cluster 2 | -3.3 (-6.3;-0.26) | 140 (-236;516) |

*β: regression coefficient from linear regression, representing the mean difference relative to the reference cluster.*

# Example of R code

⚠️ The following code snippets are provided solely for illustrative purposes and are not intended to be executed as-is.

**library**(dplyr)
**library**(tidyr)
**library**(polycor)
**library**(corrplot)
**library**(FactoMineR)
**library**(tableone)

**Data management**

#In our database, we classified exposure in several classes depending on detection result. So we had for each pollutant two, three or four classes

#Fictive Table example :

| ID | Age | Breastfeeding | DDE_RESULT_hair | DDE_RESULT_serum | DDE_RESULT_CSF | TCP_y_RESULT_hair |
| --- | --- | --- | --- | --- | --- | --- |
| 1 | 3.2 | Yes | 3 | 4 | 2 | 3 |
| 2 | 1 | Yes | 3 | NA | NA | 2 |
| 3 | 10 | No | NA | 1 | 1 | NA |
| 4 | 8 | NA | 2 | 1 | 1 | 1 |
| … | … | … | … | … | … | … |

**Correlation**

#We used polychoric correlation for categorial (ordinal) data. We extracted database for each matrice, and for each matrix match (hair-serum, serum-CSF, serum-hair-CSF…)

#Example for correlations between pesticides in hair :

polychor_hair <- **hetcor**(hair_data)
matrice_hair <- polycor_hair**$**correlations

**corrplot**(matrice_hair, method = "color", type = "upper",
 col = **colorRampPalette**(**c**("blue", "white", "red"))(200),
 addCoef.col = "black", number.cex = 0.9,
 tl.cex=1,
 tl.col="black")

**Clustering**

#We select variables including all results in hair, and other factors Difference between factor and numeric variables

data_hair <- data **%>%** dplyr**::select**(**contains**("RESULT_hair"),
 age_continuous,weight_continuous,
 age_cat,weight_cat,
 position,sex,breastfeeding,Fdep,prematurity)

data_hair <- data_hair **%>%**
 **mutate**(**across**(**-c**(age_continuous, weight_continuous), as.factor))

**class**(data_hair**$**age_continuous)
**class**(data_hair**$**age_cat)

#MCA on exposure data only ! “quali.sup” correspond to sup data (not pesticide exposure) in factor (identify column) “quanti.sup” correspond to sup data in numeric

?mca
acm <- **MCA**(data_hair,quali.sup = 15**:**21,quanti.sup = 13**:**14, graph = FALSE)
**summary**(acm)
**plot**(acm)
**plot**(acm,choix="var", invisible=**c**("quali.sup",invisible="quanti.sup"))

#Clustering from MCA results

#“clust=-1” correspond to an automatic number of cluster (it is often the best number, but you should try several number) Trying different axes allow to identify distribution in several dimensions

res.hcpc = **HCPC**(acm,nb.clust=**-**1)
**summary**(res.hcpc)
**plot**(res.hcpc,axes=**c**(1,2),choice="map",draw.tree=FALSE)
**plot**(res.hcpc,axes=**c**(2,3),choice="map",draw.tree=FALSE)
**plot**(res.hcpc,axes=**c**(1,3),choice="map",draw.tree=FALSE)

#So we have our clusters from MCA and ONLY FROM EXPOSURE DATA. Sup data are added but can’t change the clustering We add all distribution by cluster in a table, with independance test

data_clust <- res.hcpc**$**data.clust
vars <- **setdiff**(**names**(data_clust), "clust")
table1 <- **CreateTableOne**(vars = vars, strata = "clust", data = data_clust)
**print**(table1, showAllLevels = TRUE, includeNA=FALSE)

#Logistic regression. Example with breastfeeding (0=no, 1=yes)

glm(breastfeeding ~ clust, data = data_clust, family = "binomial") |> tbl_regression(exponentiate = TRUE)

#Specificity with Fdep (5 categories)

polr(Fdep ~ clust, data = data_clust, method = "logistic") > tbl_regression(exponentiate = TRUE)

# References

1.	Auger N, Fraser WD, Smargiassi A, Kosatsky T. Ambient Heat and Sudden Infant Death: A Case-Crossover Study Spanning 30 Years in Montreal, Canada. Environ Health Perspect. 2015 Jul;123(7):712–6. doi:10.1289/ehp.1307960 PubMed PMID: 25748025; PubMed Central PMCID: PMC4492261.

2.	Jhun I, Mata DA, Nordio F, Lee M, Schwartz J, Zanobetti A. Ambient Temperature and Sudden Infant Death Syndrome in the United States. Epidemiol Camb Mass. 2017 Sep;28(5):728–34. doi:10.1097/EDE.0000000000000703 PubMed PMID: 28661937; PubMed Central PMCID: PMC5552234.

3.	World Health Organization. Low birth weight [Internet]. [cited 2026 Aug 21]. Available from: https://www.who.int/data/nutrition/nlis/info/low-birth-weight
